# Moyamoya Disease in a Patient With Sickle Cell Disease: A Case Report and Review of the Literature

**DOI:** 10.7759/cureus.55592

**Published:** 2024-03-05

**Authors:** Abdulrahman B Alotaibi, Hind B Alrashedi, Tayseer S Elsafi

**Affiliations:** 1 College of Medicine, Imam Mohammad Ibn Saud Islamic University, Riyadh, SAU; 2 Department of Internal Medicine, Prince Sultan Medical Military City, Riyadh, SAU

**Keywords:** genetic disease, epilepsy, sickle cell disease, cerebrovascular disorder, moyamoya disease

## Abstract

Moyamoya disease (MMD) is a relatively rare, progressively worsening steno-occlusive condition primarily characterized by a progressive narrowing of the intracranial arteries, causing hypoperfusion and consequent cerebral ischemia and infarction. This case report discusses the rare presentation of a patient who was known to have sickle cell disease and MMD. Various investigations have revealed a typical presentation of such a disease through radiological findings. Our report highlights this rare disease and its possible association with other comorbidities, as well as the medical treatment options that patients may undergo with the option of surgical treatment.

## Introduction

Moyamoya disease (MMD) is a relatively rare, progressively worsening steno-occlusive condition first described by the Japanese neurosurgeons Takeuchi and Shimizu in 1957 [1]. The disease is primarily characterized by a progressive narrowing of the intracranial portions of the distal internal carotid artery, as well as the initial proximal components of the middle cerebral artery and anterior cerebral artery. It appears to have an unknown etiology and is associated with a tendency towards cerebral ischemia and infarction. MMD most likely affects Asians, especially in Japan, with females being impacted more frequently than men, at a ratio of 1:1.8 [1-3].

Doctors typically diagnose the condition within a child's first ten years of life [2]. Reports have also indicated unilateral involvement in MMD, although it typically affects both sides of the brain in its steno-occlusive zones [4]. Patients with sickle cell disease, Down syndrome, and neurofibromatosis type I have a greater risk of MMD [5]. We present a case of an 18-year-old male with sickle cell anemia who presented with a history of stroke with left hemiparesis, followed by seizures, ultimately revealing angiographic findings suggestive of MMD.

## Case presentation

Our patient is an 18-year-old male with a known case of sickle cell anemia, epilepsy, and hypertension. He presented to the emergency department with an inability to talk for a few hours, confusion, and sleepiness. The patient was in his usual state of health until one day prior to presentation when his mother noticed that he became sleepy with difficulty waking him up and was unable to talk. The condition was associated with mood liability. At that time, the patient had been brought to the Accident & Emergency Department, and he was reassured and sent home. However, the patient did not improve, and his condition got worse.

Upon physical examination, the patient looked well and alert to place, time, and person; he was not in distress, and there was no sign of dehydration. He was conscious but sleepy, pale, and not jaundiced or cyanosed. The patient was vitally stable, with an oxygen saturation of 100% in room air, a body temperature of 36.5 °C, a heart rate of 100 beats per minute, a respiratory rate of 28 beats per minute, and a blood pressure of 116/60 mmHg.

The cardiovascular examination was normal with first and second heart sounds, no added sounds, or murmurs. The chest exam was also normal, with equal bilateral airway entry and no added sounds. The abdominal examination was soft and lax, and no organomegaly had been noted. The Glasgow Coma Scale 15/15 and neurological exam were reassuring with normal tone, power, reflexes, and sensation. However, the ear, nose, and throat examinations are clear.

At that time, the patient was admitted to the intensive care unit (ICU) when he started to have a tonic-clonic convulsion which was treated with IV diazepam. The convulsions recurred, and he developed status epilepticus due to which he was administered phenytoin, intubated, and ventilated with sedation. Meanwhile, the neurology team saw the patient, and they started him on phenobarbitone and Keppra.

The results of the complete blood count were notable for normocytic normochromic anemia with normal white blood cells and platelets (Table 1). The complete metabolic panel was consistent with hypokalemia, which was corrected and remained within the normal limit.

**Table 1:** Lab values with reference range

Test name			Results	Reference range
White blood cells (WBC)			3.0x10^9^/L	(4.5 - 11.0 x10^9^/L )
Red blood cells (RBC)			4.64 x10^12^/L	(3.80 - 5.20 x10^12^/L)
Hemoglobin (Hgb)			126 g/L	(117 - 161 g/L)
Mean corpuscular hemoglobin (MCH)			27.2 pg	(27.0 - 35.0 pg )
Mean corpuscular hemoglobin concentration (MCHC)			36.5 g/dL	(32.8-38.6 g/dL )
Platelet			110 K/uL	(100-250 K/uL )
Neutrophils %			3.34 K/uL	(2.50-6.90 K/uL)
Lymphocyte %			1.67 K/uL	(1.50-5.10 K/uL)
Monocyte %			0.24 K/uL	(0.20-0.60 K/uL)
Eosinophils %			0.00 K/uL	(0.00 - 0.80 K/uL)
Basophils %			0.02 K/uL	(0.00-0.10 K/uL)

**Figure 1:**
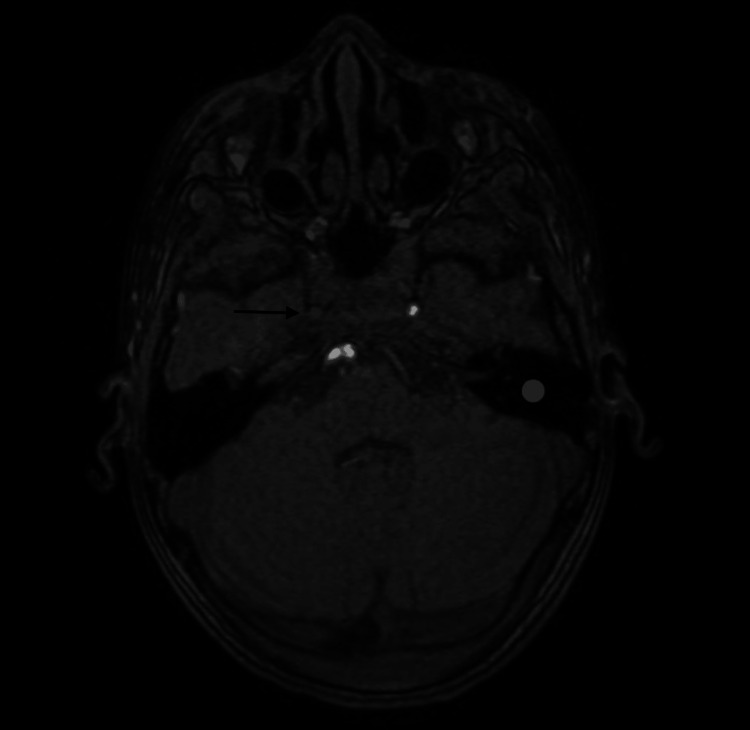
Multiple bilateral cerebral acute / subacute ischemic infarctions and right internal carotid artery occlusion (black arrow).

**Figure 2:**
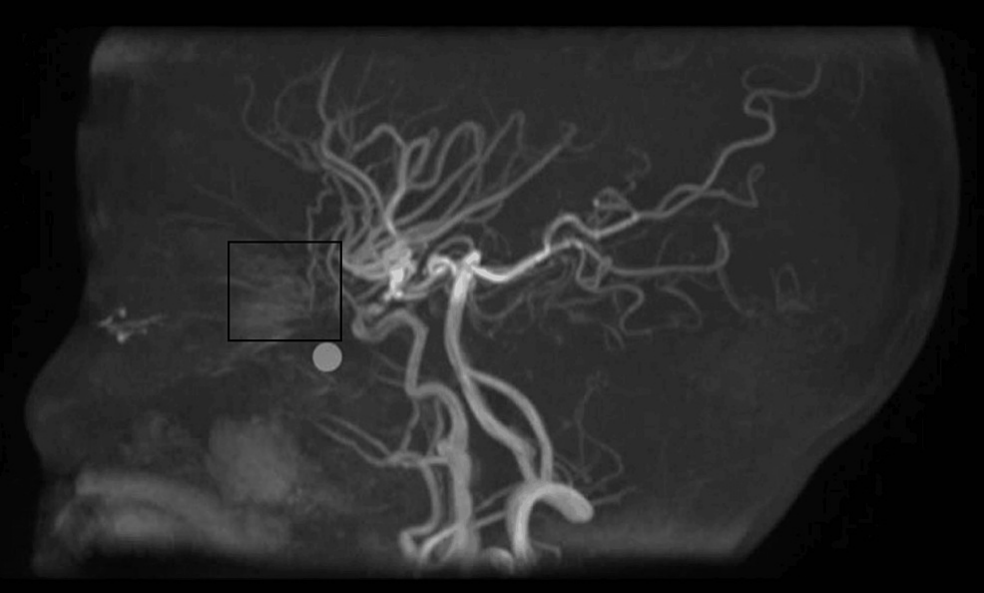
Examination of the intracranial vascular channels using 3D Time Of Flight (TOF) magnetic resonance angiography (MRA) TOF MRA shows no evidence of flow signal in the intracranial portion of the right internal carotid artery likely representing right internal carotid artery occlusion and refilling of the right middle cerebral artery and anterior cerebral artery from the contralateral side is noted. Both also show mildly attenuated caliber. "puff off smoke" phenomenon which is typical for MMD (black square).

An urgent computed tomography (CT) brain scan showed multiple ischemic lesions involving the superficial and deep gray matter of different ages, more in number on the left side. The patient went for magnetic resonance imaging (MRI) with and without contrast, which concluded multiple bilateral cerebral acute and subacute ischemic infarctions with right internal carotid artery occlusion (Figures 1-2).

## Discussion

The clinical presentation of MMD in adults and children is quite distinct [6,7]. In Japan, adults often experience hemorrhagic incidents, but children usually appear with symptoms related to ischemic injury and slowly progressing cerebral hypoperfusion [8]. However, a comprehensive series of studies including individuals with MMD have revealed that ischemic injury is prevalent in almost one-third of adult cases [8]. In MMD, the age at which the disease is first diagnosed is bimodal; in the pediatric population, this age peak occurs at age five, whereas in the adult population, the age at which it is first diagnosed occurs most frequently in the fourth decade of life [9]. MMD is recognized to be a genetically vulnerable disease, according to growing evidence from the full genome study, which indicates that in numerous East Asian countries, the Ring Finger Protein 213 (RNF 213) gene on chromosome 17q25.3 is a significant risk factor for known MMD patients [10].

The proximal parts of the intracranial internal carotid artery (ICA), anterior cerebral artery (ACA), and middle cerebral artery (MCA) are involved in the bilateral progressive stenosis of the anterior intracranial circulation caused by MMD. This results in a compensatory response that enlarges and forms collateral blood vessels for perfusion, such as the lenticulostriate arteries, giving the angiographic appearance of a "Puff of Smoke," or "Moyamoya" in Japanese. A diagnostic cerebral angiography is considered the gold standard in the image-based diagnosis of MMD. Additionally, Suzuki and Takaku initially categorized the progress of MMD into a six-stage system known as the Suzuki staging system (Table 2) [11]. However, another way to see areas of the brain affected by acute ischemia or infarction is via magnetic resonance imaging (MRI), which shows the "ivy sign" of MMD, which is basically the emergence of a hyper-intense linear high signal sulcal pattern on the Fluid Attenuation Inversion Recovery (FLAIR) sequence [12].

**Table 2:** Suzuki staging system based on the angiographic appearance of MMD.

Suzuki Stages	Angiographic appearance
Stage 1	Narrowing of the terminal internal carotid bifurcation
Stage 2	Initial development of the first Moyamoya collateral vessels at the base of the brain with dilation of the intracerebral main arteries
Stage 3	The collateral Moyamoya vessels intensify, becoming more prominent, and the major trunks of the anterior circulation become severelv stenotic and start to occlude
Stage 4	Posterior cerebral arteries become occluded, moyamoya vessels start to diminish, and collaterals from the external carotid arteries begin to form
Stage 5	Moyamoya collateral vessels begin to completely disappear, and the extracranial collaterals become more and more prominent
Stage 6	Disappearance of the moyamoya collaterals and major named cerebral arteries; the cerebral hemispheres receive blood almost exclusively from abnormal external carotid anastomosis

There is no established medical intervention that can halt the progression of MMD at this point. All medical interventions, like taking antiplatelet drugs to lower the risk of thrombus formation as cerebral arteries slowly close off, are meant to stop the disease from getting worse in the future. The implantation of endovascular interventional stents has not been able to stop the progression of MMD or prevent ischemic episodes in the future [13]. The best course of treatment for MMD is surgical revascularization, either direct or indirect. The purposes of surgical revascularization are to stop stroke and give under-perfused areas of the brain enough blood flow thereafter [1].

A surgery called the "ECA-to-ICA bypass" skips the lower parts of the external carotid artery (ECA), like the superficial temporal artery (STA) or the ophthalmic artery (OA), and connects directly to the lower parts of the internal carotid artery (ICA), like the M4 segment of the MCA. This restores blood flow to a part of the brain parenchyma that was previously under-perfused. A frequent variation of the operation is the “STA-MCA bypass," which makes a cranial defect to facilitate direct surgical anastomoses between the STA and the distal MCA (often an M4 segment). In cases where the STA is not a feasible choice due to factors including congenital hypoplasticity or injury from a previous craniotomy, an anastomosis with the M4 segment can be created via either the OA or middle meningeal artery (MMA). This part represents the direct route of surgical revascularization, which encompasses a risk of mortality ranging from 0.6% to 4.4% based on the preoperative Suzuki grade and the patient's disease severity [14].

On the other hand, all indirect approaches rely on the pure physiology of angiogenesis, which is a complex biochemical process that arises in response to wound healing and enables connections and anastomosis to grow between neighboring damaged blood vessels. Many angiogenic signaling molecules, which are essential for collateral neovascularization, are upregulated in MMD. These include fibroblast growth factor (FGF), transforming growth factor beta (TGF-β), angiopoietin-1 (Ang1), and neuropilin-1 (NRP-1) [15].

The advantages and benefits of indirect techniques are vast, but it may take months or years. Direct interventions may be more beneficial in an advanced and progressive disease condition because they immediately restore circulatory supply to parts of the cortex that are hypoperfused. One study evaluating the benefits of direct versus indirect bypass reveals that, when used early during MMD, indirect techniques provide a better clinical outcome. Compared to patients receiving conservative management with monitoring and medication therapy, individuals who have early-stage indirect revascularization surgery have a decreased overall incidence of stroke [16].

## Conclusions

MMD is a progressive and chronic illness, and while there are no viable endovascular or medical treatments for MMD, the disease can be halted surgically. No matter what age, both direct and indirect bypass surgeries that connect arteries outside the brain to those inside the brain have been shown to stop ischemic and hemorrhagic strokes. Lower Suzuki angiographic stages are crucial for intervention as they prevent the patient from becoming chronically disabled due to recurrent ischemia and hemorrhagic episodes. A subsequent study is currently investigating the underlying pathophysiological cause of MMD and could lead to the identification of potentially useful endovascular or pharmaceutical therapies. However, early surgical revascularization remains the only viable therapeutic option available.
